# Challenges and complexities in evaluating severe acute respiratory syndrome coronavirus 2 molecular diagnostics during the COVID-19 pandemic

**DOI:** 10.4102/ajlm.v11i1.1429

**Published:** 2022-04-26

**Authors:** Lesley E. Scott, Lara D. Noble, Ashika Singh-Moodley, Trish Kahamba, Diana R. Hardie, Wolfgang Preiser, Wendy S. Stevens

**Affiliations:** 1 Department of Molecular Medicine and Haematology, School of Pathology, Faculty of Health Sciences, University of the Witwatersrand, Johannesburg, South Africa; 2 National Priority Programme, National Laboratory Services, Johannesburg, South Africa; 3 Division of Medical Virology, Faculty of Health Sciences, University of Cape Town, Cape Town, South Africa; 4 Division of Medical Virology, Faculty of Medicine and Health Sciences, Stellenbosch University, Cape Town, South Africa; 5 National Health Laboratory Service, Cape Town, South Africa

## Background

Severe acute respiratory syndrome coronavirus 2 (SARS-CoV-2), the causative agent of coronavirus disease 2019 (COVID-19), was first identified in Wuhan, China, in December 2019.^1^ Since the World Health Organization (WHO) declared COVID-19 a global pandemic on 11 March 2020,^2^ more than 280 million cases of infection and over 5 million deaths had been reported globally by 31 December 2021.^3^ The primary method of diagnosing infection with SARS-CoV-2 is by nucleic acid amplification technology (NAAT). Diagnostic assays have become available at an impressive rate, with a total of 514 molecular assays listed by December 2021.^4^ Regulatory bodies in Australia, Brazil, Canada, Europe, Japan and the United States, and organisations such as the WHO, the United States Food and Drug Administration, the Foundation for Innovative New Diagnostics (FIND), and the United States Centers for Disease Control and Prevention (CDC) provide ongoing updates on assay performance. Many assays, however, remain unavailable in certain geographic regions; in-house assays are in use that are not accessible to these evaluation bodies, and the ongoing emergency use need has meant that national programmes have had to perform their own evaluations. Furthermore, in mid-2020, limited guidance was available for performing such evaluations and equally limited guidance existed on acceptance criteria or patient use cases.^5^ The FIND had verified 21 NAAT assay manufacturer claims^6^ (< 8% of available assays by July 2020), and the WHO target product profile (TPP) version 0.1 was only released in August 2020.^7^ Through emergency validations in South Africa, numerous complexities of molecular testing were quickly realised. Many of these align with those described elsewhere,^8,9^ but additional difficulties arose due to drastic local lockdown measures, and the experience of using assays during lockdown yielded several relevant insights.

## Complexities of NAAT selection

**FIGURE 1:**
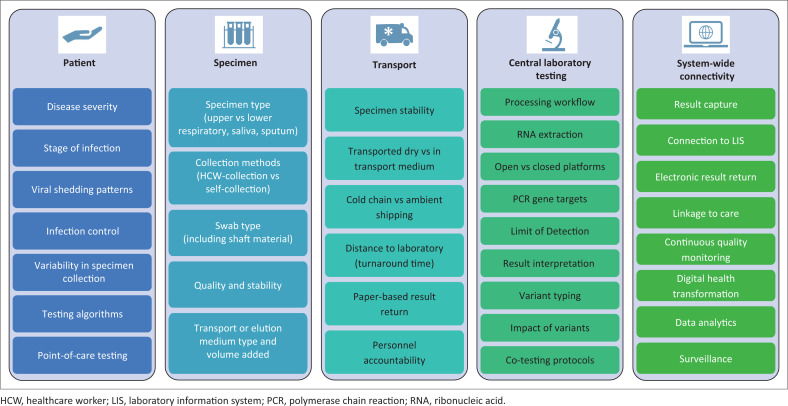
Considerations for severe acute respiratory syndrome coronavirus 2 molecular assays across the pathology value chain.

The entire pathology value chain needs to be considered when selecting suitable NAATs. Each step contributes to meaningful and valid test results: patient sampling, specimen type, specimen collection and handling (stability), viral RNA purification, amplification and detection, and result interpretation (Figure 1). Specimen collection and handling challenges include swab type, collection site, collection and pre-treatment methods, and transport. Swabs are manufactured from different materials (cotton, nylon, polyester, rayon and foam) and may be spun or flocked.^10,11^ Thus, swabs differ in absorption properties, viral capture and release. Swab shafts may be made from wood, plastic or aluminium, and some of these materials are incompatible with certain collection and laboratory techniques (e.g. wooden shafts are unsuitable for paediatric sampling and may inhibit NAAT).^10^ Commonly used swab collection sites are the nasopharynx, mid-turbinate region, nasal cavity or oropharynx, but the method used for a particular specimen is often not documented or made known to a testing laboratory. Alternative specimen types include saliva, gargle, sputum and faecal material. Furthermore, multiple swabs may be combined in a single testing vial. After collection, the swabs may be transported dry or in plain saline, phosphate-buffered saline, or various preservation media (e.g. viral transport media). The medium may vary in volume (1 mL – 3 mL), impacting viral concentration. Specimens are transported at 2 °C – 8°C or at ambient temperature, which, in some settings, means high humidity and temperature. Certain testing laboratories insist on inactivation of viral swabs for biosafety, which could include chemical or heat pre-treatment.

Viral RNA extraction (or purification) is prone to variability. The majority of RNA purification methods rely on automated magnetic bead extraction performed on automated platforms (e.g. NucliSENS^®^ EasyMag^®^ [bioMérieux, Johannesburg, South Africa]). These differ in processing capacity, input volume, purified RNA elution yield and turnaround time (TAT). Manual methods range from spin columns (e.g. RNeasy Mini Spin [Qiagen, Hilden, Germany]), which rely on centrifugation, to crude approaches that are used where no extraction instrumentation is available. The latter include direct lysis-to-polymerase chain reaction (PCR) kits (e.g. Lyra® Direct SARS-CoV-2 Assay [Quidel^®^, San Diego, California, United States]), proprietary lysis buffers (e.g. Bosphore EX-Tract Dry Swab RNA [Anatolia Geneworks, Istanbul, Turkey]) or simply heat (where rapid sample preparation is needed).^12^ These procedures aim to disrupt viral particles to release RNA and inactivate inhibitory enzymes. Added variables with the lysis approach could be a specimen’s exposure time to the buffer and buffer compatibility with PCR. The primary specimen input testing volume also differs between technologies: 300 µL added directly to an Xpert^®^ Xpress^®^ SARS-CoV-2 cartridge (Cepheid, Sunnyvale, California, United States), 400 µL to the cobas^®^ SARS-CoV-2 assay (Roche, Basel, Switzer;and) and 500 µL to the RealTi*m*e SARS-CoV-2 assay (Abbott, Chicago, Illinois, United States). Similarly, most RNA extraction platforms require input of anywhere between 100 µL and 300 µL of raw specimen. Furthermore, for a direct lysis-to-PCR assay, input volume can range from 10 µL preservation buffer (NaGene [Diagnóstica Longwood, Zaragoza, Spain]) to 30 µL raw specimen (SMARTCHEK^®^), and ideally the patient swab should be added directly to the proprietary medium.

The NAATs used are also variable. They are performed on a variety of platforms that may be closed (dedicated) (e.g. m2000^®^ [Abbott, Chicago, Illinois, United States]) or open (‘plug-and-play’ approach subsequent to extraction using different systems and instruments). The latter requires compatibility with real-time thermocycler instruments (e.g. CFX96 Touch™ [BioRad, Hercules, California, United States], QuantStudio [Thermo Fisher Scientific, Waltham, Massachusetts, United States]). Throughput ranges from a single test at a time (e.g. GeneXpert^®^ [Cepheid, Sunnyvale, California, United States]) to 1000–1500/day (e.g. Cobas^®^ [Roche, Basel, Switzerland]). Many of the open-platform thermocyclers use 96-well plates and may require specimen batching. As with specimen input volume, the RNA input volume also varies between assays (e.g. 8 µL for the AllPlex SARS-CoV-2 assay [SeeGene, Seoul, South Korea], 10 µL for the TaqPath™ SARS-CoV-2 assay [Thermo Fisher Scientific, Waltham, Massachusetts, United States], and 14 µL for the PerkinElmer™ SARS-CoV-2 RT-qPCR Reagent Kit CE-IVD [PerkinElmer™, Waltham, Massachusetts, United States]. A number of novel assays have RNA input volumes as low as 5 µL. The differences in volume for extraction or PCR may influence theoretical limits of detection.

In addition to these complexities, the SARS-CoV-2 genes (envelope [*E*], nucleocapsid [*N*], spike [*S*], membrane [*M*], open reading frames 1a and 1b [*ORF1ab*], RNAse-dependent RNA polymerase [*RdRP*]) or combination of genes targeted by NAAT are inconsistent. These have been reported to affect overall assay sensitivity and specificity,^13,14^ and may also be impacted by viral genetic evolution that can affect gene targets.^15^ Furthermore, there is the complex layer of different diagnostic algorithms implemented within countries that often change over time. For example, some settings report specimen results based on a single gene target, while others require two or more gene targets or result confirmation by a second NAAT method using a different gene target. Automated software algorithms are not always available for all PCR platforms, and result analysis may require skilled user input (e.g. visual interpretation of amplification curves or adjustment of threshold settings and thus cycle thresholds [Ct]). This adds another, potentially subjective, layer of variability.

## Challenges of robust and rapid NAAT evaluations

The complex variables described above come to the fore when clear, comparative standardised evaluations are conducted. Such evaluations generally had to be performed in parallel with managing COVID-19 testing emergencies. In our case, the most challenging issue experienced early in the pandemic was access to sufficient numbers of relevant clinical specimens available for inclusion in evaluation challenge panels. The volume of extracted RNA per specimen was often only sufficient to test a limited number of NAATs. Thus, constant panel ‘manufacture’ was required, with the potential to introduce variability (e.g. different specimens) across evaluations. The sample size, the numbers of positive and negative panel specimens, and the range in viral concentration (high, medium or low) of challenge specimens may influence sensitivity scores. The last addresses the need for assays to correctly identify differences in patient risk stratification: while there does not appear to be a difference in median Ct between symptomatic and asymptomatic patients,^16^ a lower Ct (higher viral load [VL]) in real-time PCR assays may indicate increased virus transmissibility^16,17^ and may impact patient outcomes.^17,18^

The impact of SARS-CoV-2 variants on technologies^15^ should also be considered, as specimens selected from waves of infection driven by different variants could affect the technology (e.g. *S* gene target failure of the TaqPath SARS-CoV-2 assay [Thermo Fisher Scientific, Waltham, Massachusetts, United States] with the Alpha^19^ and Omicron^20^ variants). These challenges contribute to generating variability in the assay performance outcome and may make the difference between accepting different assays and criteria. False negative results can arise from low levels of virus due to patient or sample characteristics (i.e. either biologically or artefactually), degraded viral RNA, viral genetic variation or presence of inhibitors. False positive results can arise from inaccurate Ct threshold settings, and contamination during processing. The correct placement of NAAT technologies should also be considered. A less sensitive assay may be valuable in settings that receive specimens from patients with high VL concentrations (low Ct values), that is, emergency settings or referral centres. In contrast, community or mobile screening and testing sites will require assays with greater sensitivity.

In addition to clinical specimens, several types of reference materials (plasmids, biomimetic standards, or viable, inactivated or lysed virus) have become commercially available (e.g. AccuPlex™ SARS-CoV-2 Reference Material Kit [SeraCare, Milford, Massachusetts, United States]), although these may not always be compatible with NAAT primer or probe sequences. Access to locally manufactured SARS-CoV-2 viral culture supernatant is an asset to expanding an evaluation panel, independent of NAAT target genes. Culture dilutions may be used to measure assay precision, linearity and, potentially, the limit of detection. Another limitation is often kit size, where only a limited number of specimens can be tested per kit supplied or where single-plex PCR assays limit the number of specimens in a test run (e.g. single-plex with 3 gene targets can only assess 32 specimens per 96-well RNA extraction plate including controls).

Other considerations determined during performance evaluation are assay ease of use, time to reportable result and throughput. General considerations, often listed in a TPP are: testing footprint, number of testing steps, operator skills required to perform the test, minimum pipetting volume, biosafety requirements, internal controls, positive and negative batch controls, reagent storage stability, reagent reconstitution required, training needs, maintenance, additional consumables and connectivity options, to name a few. Moreover, it is important to select suitable comparator technology, a limitation also described by Axell-House et al.^21^ This may be based on WHO recommendations, or, under emergency use, current in-county methodology already approved for standard of care (SOC). In our case, at least seven SOC technologies are in use to report patient results across South Africa, and residual specimens from these assays were available for challenge panels. The goal of performance evaluation is to determine whether a new method or technology is as good as SOC and use thereof will not alter patient care. However, this does pose a challenge if SOC specimens are in a different format or RNA degrades during specimen storage. Our group obtained institutional review board approval to use residual patient specimens for evaluations with an informed consent waiver. Where fresh or paired specimens were needed, a full clinical trial was required with informed consent, which increased the time needed and cost of evaluations.

Once an evaluation is completed, method comparison involves determining accuracy (sensitivity and specificity) or agreement, precision (reproducibility), linearity and limit of detection. Recommendations are made based on analytical performance, as well as ease of use. In the absence of acceptance criteria guidelines, we investigated these parameters for an initial 24 assays using a standardised evaluation protocol approach as described in Table 1. Over and above the already mentioned complexities and challenges, it became apparent that single method comparison acceptance criteria might not be applicable to all assays. We identified four types or categories of assays submitted for evaluation: (1) closed systems (incorporating RNA extraction and NAAT), (2) standalone PCR kits to be performed off already purified RNA, (3) direct lyse-to-PCR, where no front-end RNA purification or extraction is available or where there is a need for rapid testing, and (4) standalone RNA extraction or purification kits. The 24 assays were among groups 1 (*n* = 9), 2 (*n* = 12) and 3 (*n* = 3), with the predominant viral gene targets being *N* (35.0%), followed by *ORF1ab* (25.0%), *E* (20%), *S* (10.0%) and *RdRp* (10.0%). A sensitivity score of > 90% was achieved by 62.5% (15/24) of assays and > 95% sensitivity achieved by 37.5% (9/24) compared to SOC. Those that employ direct lyse-to-PCR (group 3) were below the acceptance criteria by at least a 20% drop in median sensitivity (72.0%) compared to the median sensitivity in group 1 (95%) and group 2 (92.0%). The clinical specimens used in these assays’ evaluation panels covered a range in SARS-CoV-2 VL, with a median target Ct = 27. Group 3 performed well on specimens with high VL (Ct < 30), and hence another challenge is not only in the choice of range in clinical specimens being evaluated, but potentially the need to apply different acceptance criteria, bearing in mind that group 3 assays are designed for use where no front-end RNA extraction (or rapid) system is available. The reduced sensitivity needs to be weighed against the rapid availability of a result which would likely identify patients who are newly symptomatic and at the height of being infectious. Therefore, the diagnostic dilemma facing regulators and end users could be ‘no available test’ or ‘specimens routed a further distance (with ensuing longer TAT) to a testing laboratory with access to front-end RNA extraction technology’, or a ‘less sensitive test with potentially shorter TAT and greater clinical relevance’.

**TABLE 1:** Key evaluation features of a protocol applied under emergency use and acceptance criteria.

Evaluation feature	Evaluation material	Acceptance criteria
Precision (reproducibility): at least intra-variability (within one run, within 1 day, within one instrument across a range of dilutions performed in triplicate or more)	Viral culture supernatant, spiked swabs, reference material (commercially available), plasmids or biomimetic standards, or diluted residual patient specimen	Standard deviation of the cycle threshold < 3.0 (i.e. < 1 log viral concentration). Coefficient of variation < 5%
Limit of detection (can be measured during the precision evaluation) and linearity (across relevant viral concentrations)	Linear dilution range of residual patient specimen (or quantified reference material) across a range in viral load (cycle threshold)	< 300 cp/mL or equivalent determined by reference material categories (may increase for lyse-to-polymerase chain reaction tests)
Accuracy (agreement) across a clinically relevant range of viral concentrations (to accommodate all patient risk stratification)	Residual clinical specimens (swabs in phosphate-buffered saline or viral transport media, extracted RNA) Reference and test technology preferably performed within 24 h of each other Residual specimens may be stored at –70 °C if aliquoted to minimise number of thaw cycles	An overall sensitivity > 95% shows an assay with good performance in identifying SARS-CoV-2 at all ranges of viral concentration and therefore patient’s course of infection An overall sensitivity > 90% shows an assay with acceptable performance identifying SARS-CoV-2 100% specificity
Assay robustness	Number of errors, invalids	< 3% invalid rate, < 1% error
Ease of use	Based on a target product profile (e.g. www.sahpra.org.za^24^ and, more recently, WHO^7^)	Based on a target product profile (e.g. www.sahpra.org.za^24^ and, more recently, WHO^7^)

SARS-CoV-2, severe acute respiratory syndrome coronavirus 2.

## Conclusion

Selecting clinically relevant, laboratory-compatible and well-performing SARS-CoV-2 NAAT assays or systems for patient care is complex and subject to several challenges, as also highlighted elsewhere.^9,22^ Current evaluation protocols are not robustly performed under emergency circumstances,^21^ and performance acceptance criteria and technology placement may require flexibility.^23^ Assays themselves have also improved with time. Added layers of complexity that we experienced were donated tests that did not always follow required regulatory pathways prior to implementation, and suppliers requesting evaluation from multiple laboratories in the hope of improving their performance score. However, having a new test ready for use is only half the battle, and still requires rapid implementation and scale-up, with further factors requiring investigation such as cost, supply chain management, training, quality assessment, interfacing to existing laboratory information systems, compatibility to existing testing landscapes, continuous quality monitoring and post-market surveillance, to name a few.
